# Characterisation of General Adult Psychiatry (GAP) Inpatients

**DOI:** 10.1192/bjo.2026.11188

**Published:** 2026-06-30

**Authors:** Hira Ahmad, David Hayward, Donald MacIntyre, Douglas Steele

**Affiliations:** 1 NHS Lothian, Edinburgh, United Kingdom; 2 University of Edinburgh, Edinburgh, United Kingdom; 3 University of Dundee, Dundee, United Kingdom; 4 NHS Tayside, Dundee, United Kingdom

## Abstract

**Aims:**

GAP is the largest psychiatric specialty, but most treatment evidence is generated in other populations, or excludes participants with severe conditions, comorbidities and treatment-resistance–essentially omitting most GAP inpatients. Consequently, the published evidence may have limited relevance.

Aimswere to characteriseGAP inpatients–diagnoses, severity, treatment-resistance, comorbidities, and legal status–to benchmark and inform the development of a framework to meta-analyse publications to test how accurately they reflect real-world practice.

**Methods:**

We characterised consecutive admissions to a GAP ward using the measures usually employed in psychiatric publications. Data included demographics, Brief Psychiatric Rating Scale (BPRS), Inventory of Depressive Symptomatology (IDS-SR), Personality Disorder Severity (PDS-ICD-11), Generalised Anxiety Disorder scale (GAD-7), Substance Use scale (TAPS), and medical abnormalities and comorbidities.

**Results:**

N=65 (‘GAP-65’, 11 declined/could not be consented). **Psychosis Group** N=32: 60% treatment-resistant (clozapine protocol), severe psychosis (BPRS mean 58.2), very severe depression (IDS-SR mean 39.4), smoking, alcohol, and cardiometabolic disease frequent. **Bipolar Mania Group** N=5: Severe mania (YMRS mean 39.6), 60% obese, 50% hypertensive, and frequent metabolic abnormalities (DM II, prolactin elevation). **Bipolar Depression Group** N=2: All treatment-resistant (Massachusetts General Hospital Staging), 50% DM II and hypertensive. **Unipolar Depression Group** N=9: Very severe depression (IDS-SR mean 50.6), 57.1% treatment-resistant, 38% obese, 71% hypertensive, and high rates of cardiometabolic and respiratory illness. **Axis II Group** N=15: 80% PD, 93.3% suicidal ideation, physical comorbidities less common but mean BMI 30.2.

**Conclusion:**

When these features are operationalised, an extremely low proportion of GAP inpatients would meet criteria for recruitment into even exemplars of contemporary academic psychiatry e.g. 1) translational neuroscience; Rupprechter et al., Brain (2020) (£4.7M, N=475)–92% participants were in the healthy or mild depression range and treatment-resistant patients were excluded c.f. GAP-65 who were all severely ill and mostly treatment-resistant. 2) treatment trials; Jog et al., JAMA Netw Open (2025) (highlighted by evidencealerts.com, including for clinical relevance)–participants were in the moderate depression range, treatment-resistant patients were excluded, and the reduction in depression scores following transcranial direct current stimulation would not move pertinent GAP-65 patients out of the severe range of illness.

Our results highlight a critical gap in psychiatric research: Studies that are highly cited and influential do not adequately represent the severity and complexity of typical GAP inpatients. Correspondingly, we propose a systematic review and meta-analysis of clinically important features of GAP inpatients to evaluate the extent to which psychiatric research represents real-world practice.

